# Experimental Research on the Energy Evolution of Concrete under Impact Loading

**DOI:** 10.3390/ma16145140

**Published:** 2023-07-21

**Authors:** Huan Wang, Faning Dang, Jie Ren, Yanjun Li, Lin Zhu

**Affiliations:** 1 School of Civil Engineering and Architecture, Xi’an University of Technology, Xi’an 710048, China; 2 State Key Laboratory of Eco-Hydraulics in Northwest Arid Region, Xi’an University of Technology, Xi’an 710048, China; 3 Shaanxi Key Laboratory of Loess Mechanics and Engineering, Xi’an University of Technology, Xi’an 710048, China; 4 College of Art and Design, Shaanxi University of Science & Technology, Xi’an 710061, China

**Keywords:** concrete, SHPB experiment, energy evolution, strain rate effect, hysteresis of energy release

## Abstract

This paper presents an experimental study on the dynamic strength of concrete by using a split Hopkinson pressure bar. The stress–strain relationship and fragmentation degree of concrete were analyzed. The change process of the incident energy, reflection energy, transmission energy and consumption energy of concrete was calculated. The corresponding relationship between the variation of each energy and the stress state of concrete was studied. The law of energy evolution during the concrete fracture process was determined and the mechanism of concrete dynamic strength increase was revealed from the perspective of energy. The results show that the higher the strain rate, the higher the fragmentation degree of concrete, the smaller the grain diameter of fragments, the easier cracks are to pass directly through the aggregate, and the more regular the fragment shape. The change process of increasing amplitude of concrete consumption energy can reflect four mechanical states of concrete: stress increase, stress slow releasing, stress rapid releasing, and return-to-zero stress. Since the increase in reflected energy does not increase immediately with the increase in strain rate, it leads to the hysteresis of energy release in concrete, resulting in an increase in the dynamic strength of concrete.

## 1. Introduction

As the most common building material, concrete has been widely used in various industries, such as urban construction, national defense, water conservancy, etc. As the international situation changes, the probability of buildings being subjected to missiles, explosives and other impact and explosive loads increase rapidly. The mechanical properties of concrete under dynamic loading are quite different from those under static loading, therefore, only when the mechanical properties of concrete under impact loading are accurately described can we more accurately predict the stability of buildings under impact loading.

The most common method for studying the dynamic mechanical properties of concrete under impact loading is to use a split Hopkinson pressure bar (SHPB) [1,2] apparatus, which was first used by Zielinski et al. [3] to determine the dynamic compressive strength of concrete. Since then, scholars have conducted numerous studies on the dynamic mechanical properties of concrete using the SHPB test from different aspects [4,5], such as the influence of aggregate ratio [6], water-cement ratio [7], static strength [8,9], temperature [10,11], confining pressure [12] and other factors on the dynamic strength of concrete, among which the most basic and core issue is the study on the mechanism of dynamic strength increase in concrete. Through experimental observations and theoretical assumptions, Rossi et al. [13] thought that the improvement of the mechanical properties of concrete at high strain rates could be explained by the interaction between the Stefan effect and the cracking process of the material, that is, the viscous effect of free water. This method is only a bold hypothesis, which has not yet been verified by experiments. Li et al. [14], Lu et al. [15] and Zhang et al. [16] believed that the dynamic strength increase in concrete was caused by the combined action of the inherent rate sensitivity of the material and the transversal inertial effect and that concrete changed from a one-dimensional stress state to a one-dimensional strain state when subjected to an impact load. On this basis, Hao et al. [17] further studied the effect of the transversal inertia effect on the dynamic strength of concrete. Pan Feng and Dang Faning [18] considered that the dynamic strength increase in concrete was caused by the combined action of the inertial effect and the fact that high-strength aggregate passed through the cracks. Chen et al. [19] held that concrete material did not have a strain-rate effect, whose increase in dynamic strength was entirely due to the transverse inertia effect.

The main reason why the mechanism of the dynamic strength increase in concrete has not been unified is the limitations of existing test methods. Studying the strength and deformation characteristics alone cannot effectively reveal the change mechanism of dynamic mechanical properties, and the stress–strain relationship describing the specific mechanical state of concrete is only one aspect of the thermodynamic state of concrete [20]. The theory and research indicate that energy evolution is an essential physical process and plays an important role in describing the deformation and failure processes of materials [21], such as crack propagation, concrete failure, etc. Studying the dynamic mechanical properties of concrete from the perspective of energy will help reveal the mechanism of dynamic strength increase.

At present, the study of concrete in terms of energy mainly includes two aspects: fracture energy and energy absorption rate. In fracture mechanics, it is assumed that the energy required for fracture propagation is converted from the energy released by the system [22]. Therefore, scholars study the mechanical characteristics of concrete cracking through fracture energy and discuss the influences of specimen size [23], aggregates [24] on fracture energy. In terms of energy absorption rate, the energy absorption capacity of concrete under dynamic loading is mainly studied; for example, Lu et al. [25] used the SHPB apparatus to study the influence of different aggregate particles on energy absorption capacity of concrete, analyzed the relationship between average incident energy change rate and energy absorption rate under different granular materials and particle volume fraction and considered that the increase in energy absorption rate of concrete was caused by the more uniform stress distributions in the material. By calculating the energy absorption ratio, Bai et al. [26] studied the energy absorption capacity of concrete at different strain rates, and the more suitable aggregate was determined.

Although some previous studies on the dynamic mechanical characteristics of concrete from the perspective of energy have been carried out, the existing research emphasizes the total amount of energy absorption, and there are few studies on the process of energy conversion. However, the improvement in the dynamic strength of concrete is closely related to the failure process of concrete. Therefore, studying the law of energy evolution in the process of concrete dynamic failure and the relationship between energy evolution and dynamic strength are of great significance for revealing the mechanism of concrete dynamic strength increase.

In this paper, from the perspective of energy, the stress–strain relationship of concrete under different strain rates was studied by using the SHPB apparatus, and the effect of the strain rate on the crushing model and fragmentation distribution of concrete was analyzed. The energy analysis method is used to study the energy evolution process of concrete under dynamic loading and analyze the relationship between the energy evolution process and the stress–strain change process of concrete. Finally, by comparing the energy evolution process of concrete under different strain rates, the mechanism of the dynamic strength increase in concrete is revealed from the perspective of energy.

## 2. Tests on Concrete Using SHPB

### 2.1. Experimental Principle and Energy Analysis Method

**Figure 1:**
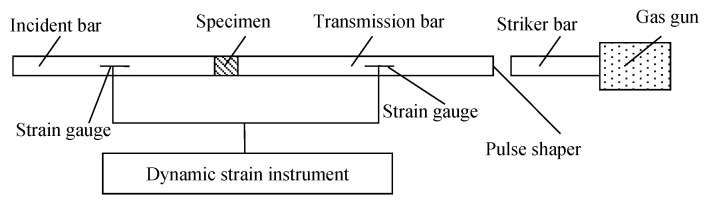
Schematic of split Hopkinson pressure bar apparatus.

Considering the heterogeneity of concrete materials, the SHPB apparatus with a small diameter is not suitable for concrete material experiments. Therefore, impact compression tests on concrete are carried out by a SHPB apparatus with a diameter of 100 mm in this paper. The effectiveness of the SHPB experiment is based on the following two assumptions: (1) the propagation of the stress wave in the bar is a one-dimensional stress wave; (2) the stress of the specimen is evenly distributed along its length. A schematic of the SHPB is shown in Figure 1 [8,10,11]. The specimen under investigation is sandwiched between the incident bar and transmission bar. The striker is launched by the gas gun and impacts the pulse shaper. This impact produces a one-dimensional stress wave that travels through the incident bar. During the experiment, the stress wave propagates along the incident bar and the specimen, which is sandwiched between the bars, before finally propagating along the transmission bar. The strain gauges that pass the strain to the hyperdynamic strain meter are attached to the incident bar and transmission bar to record the curve of experimental process, and then the incident, reflected and transmitted strain pulses ($\varepsilon_{i}$, $\varepsilon_{r}$, $\varepsilon_{t}$) can be obtained. According to the two basic assumptions of SHPB experiments, the average stress $\sigma_{s}$, strain ε and strain rate $\dot{\varepsilon }$ of the specimen can be determined by the two-wave method as follows:

(1) $$\sigma_{s}\left(t\right)=\frac{EA_{0}}{A_{s}}\varepsilon_{t}$$

(2) $$\dot{\varepsilon }\left(t\right)=-\frac{2C_{0}}{l_{s}}\varepsilon_{r}$$

(3) $$\varepsilon \left(t\right)=-\frac{2C_{0}}{l_{s}}\int \varepsilon_{r}dt$$

where E, $C_{0}$ and $A_{0}$ represent the elastic modulus, wave velocity and cross-sectional area of the bars, respectively, and $A_{s}$ and $l_{s}$ denote the initial cross-sectional area and the length of the specimen, respectively.

In the SHPB impact loading process, because both the incident bar and transmission bar are made of steel, most of them are in the elastic deformation range during impact. The incident energy is divided into three parts while the bar is in contact with the specimen: the first part is the energy of reflected wave in the incident bar; the second part is the energy of transmitted wave in the transmission bar; the third part is consumption energy in specimen during the loading process, the energy mainly consumed by the cracking and the crack propagation of the specimen. During the impact, the stress amplitude generally does not exceed the elastic limit of the steel bar, and the stress–strain relationship of the bar is linear. If the strain can be obtained, the stress can be converted and the incident, transmitted and reflected waves can be integrated to calculate the incident energy $W_{i}(t)$, reflected energy $W_{r}(t)$ and transmission energy $W_{t}\left(t\right)$. The specific calculation equations are shown as follows:

(4) $$W_{i}\left(t\right)=A_{e}EC_{0}\int \varepsilon_{i}^{2}dt$$

(5) $$W_{r}\left(t\right)=A_{e}EC_{0}\int \varepsilon_{r}^{2}dt$$

(6) $$W_{t}\left(t\right)=A_{e}EC_{0}\int \varepsilon_{t}^{2}dt$$

Assuming that the energy loss at the surface between the specimen and the incident bar and the transmission bar is ignored, according to the law of energy conversation, the consumption energy $W_{s}\left(t\right)$ of the concrete specimen can be expressed as follows:

(7) $$W_{s}\left(t\right)=W_{i}\left(t\right)-W_{r}\left(t\right)-W_{t}\left(t\right)$$

### 2.2. Preparation for the Tests

**Figure 2:**
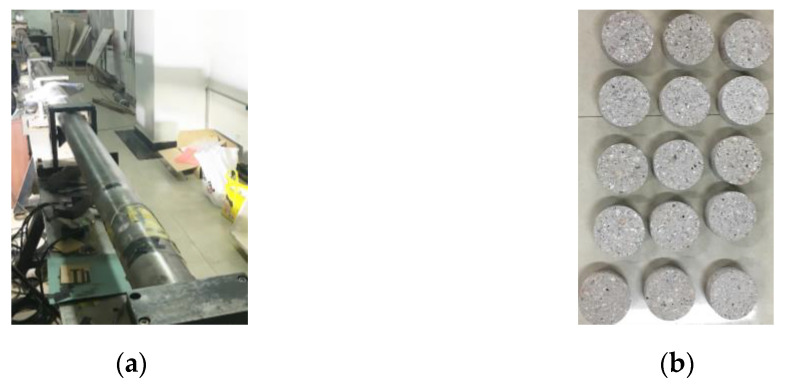
(**a**) SHPB apparatus; (**b**) Concrete specimen preparation and installation.

The loading devices of the SHPB test and concrete specimens are shown in Figure 2. In this test, normal Portland cement is used to prepare the specimens for the test. The mix proportions of concrete are shown in Table 1. Concrete whose aggregate diameter is 5 mm–20 mm with a volume ratio of 37% was taken as the research object. Concrete specimens were poured and molded into cubic molds and cured for a period of 28 days. Then, after being drilled, cut and polished, concrete specimens with a diameter of 100 mm and a length of 50 mm can be obtained.

The bars used in this SHPB system are steel bars with an elastic modulus of 210 GPa, a longitudinal wave velocity of 5200 m/s, Poisson’s ratio of 0.25 and a density of 7840 kg/m^3^. Two strain gauges with a 1.9 sensitivity coefficient and 1000 Ω electric resistance value are employed to record stress waves. The change in the strain rate can be reflected by changing the velocity of the striker. The red copper is chosen to be pulse shaper. In addition, the lengths and diameter of the incident bar are 6000 mm and 100 mm, and the length and diameter of the transmission bar are 4000 mm and 100 mm, respectively.

## 3. Results and Discussion

### 3.1. Stress–Strain Curve

**Figure 3:**
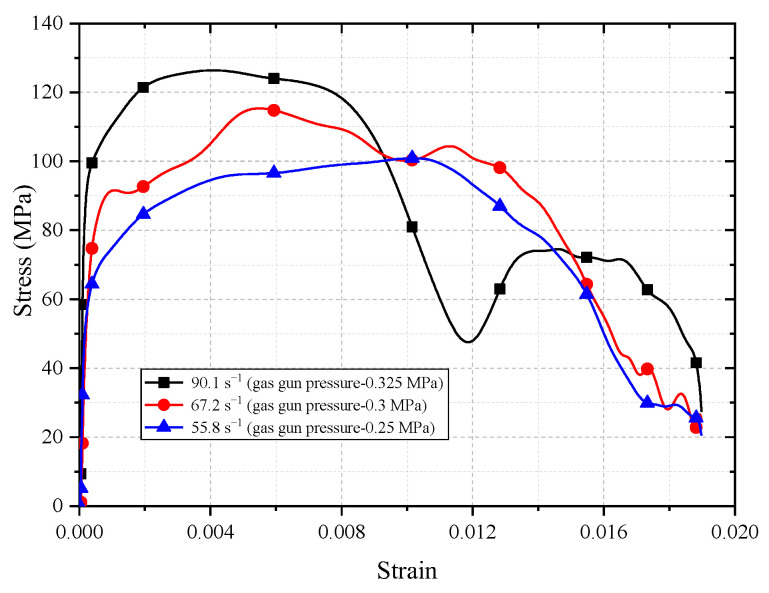
Stress–strain curves of concrete under different strain rates.

The SHPB compression test was conducted on concrete samples with an aggregate volume ratio of 37%, and the gas gun pressure was selected as 0.25 MPa, 0.3 MPa and 0.325 MPa, respectively. Because of the mesoscopic heterogeneity of concrete, the mesostructure of every specimen is different, which leads to differences in mechanical characteristics. Therefore, five tests were conducted for each pressure, and the specimen that was the closest to their average test strength was selected for study, and the stress–strain curves of concrete under different strains were obtained, as shown in Figure 3. When the strain rates were 55.8 s^−1^, 67.2 s^−1^ and 90.1 s^−1^, the dynamic compressive strength of concrete was 100.89 MPa, 115.36 MPa and 126.40 MPa, respectively. It can be seen that with the increase in loading rate and strain rates, the dynamic compression strength of concrete also increases, which is the same as the test results of a large number of scholars.

### 3.2. Analysis of Fragmentation

The failure process of concrete is as follows: the micro-cracks expand and connect to form macro-cracks, and the macro-cracks expand to form through-cracks until the concrete loses its bearing capacity. Therefore, the damage of concrete has a direct relationship with crack propagation, and the degree of damage can reflect the mechanical properties of concrete.

**Figure 4:**
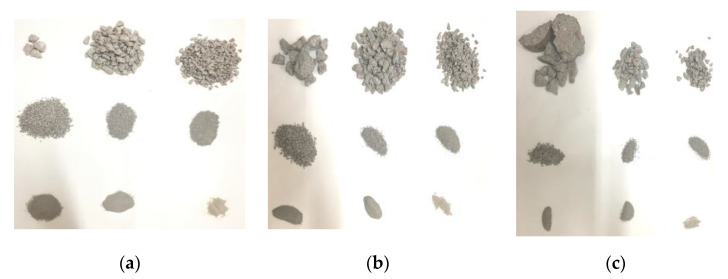
Fragmentation of concrete under different strain rates. (**a**) 90.1 s^−1^ (gas gun pressure-0.325 MPa); (**b**) 67.2 s^−1^ (gas gun pressure-0.3 MPa); (**c**) 55.8 s^−1^ (gas gun pressure-0.25 MPa).

The concrete specimens damaged in each test were collected and screened using a screening device. The fragmentation distribution after screening is shown in Figure 4. It can be found that there are still large-diameter fragments after the concrete is damaged at a strain rate of 55.8 s^−1^, and some areas are not broken along the loading direction. However, with increasing strain rate, the fragment degree of concrete becomes greater. In particular, when the strain rate is 90.1 s^−1^, there are only four pieces with a diameter of more than 20 mm, and the diameter is only slightly longer than 20 mm. It can be seen that the higher the strain rate is, the greater the fragment degree will be.

**Figure 5:**
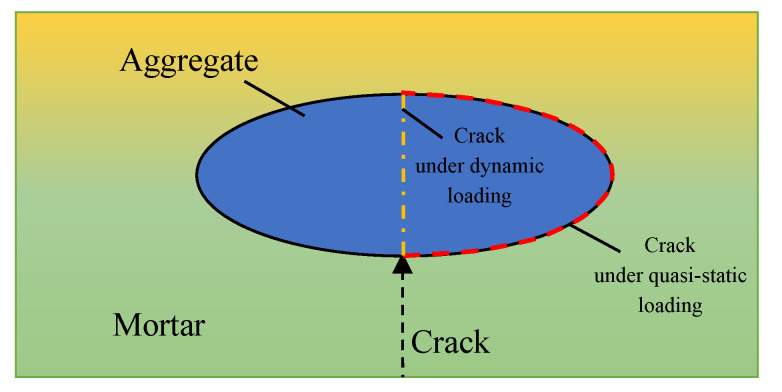
The development of the crack.

Compared with the characteristics of the cracks developing around the aggregates in the static test, no complete aggregate was found in the concrete fragments under dynamic loading; that is, in the dynamic failure process of concrete, the cracks spread through the aggregates. The main reasons are as follows: cracking will stop when it expands to the aggregate and will expand again after accumulating enough energy. There are two propagation paths: spread around the aggregate or directly through the aggregate. During quasi-static loading, due to the low loading rate, the cracks have enough time to expand, so they will propagate along the low-strength mortar. However, during dynamic loading, the loading rate and the energy input rate are high, and the time of crack propagation is short. When the energy accumulated at the tip of the crack is sufficient, the crack will directly expand along the aggregates and along the shortest path of failure, as shown in Figure 5. Therefore, it can be found that the higher the strain rate is, the more regular the shape of the damaged concrete fragments is, which is close to cuboid.

**Figure 6:**
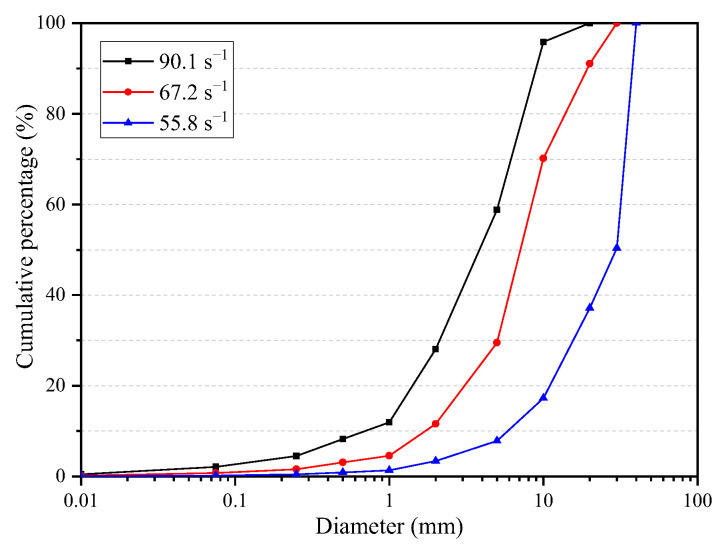
Fragmentation distribution of concrete under different strain rates.

By comparing the screening results, the distribution curve of concrete fragmentation under different strain rates is obtained in Figure 6. When the strain rate is 55.8 s^−1^, the particle size range of concrete fragments with the highest proportion is more than 20 mm, accounting for 82.7%. When the strain rate is 67.2 s^−1^, the particle size range of concrete fragments with the highest proportion is 10~20 mm, accounting for 40.7%. When the strain rate is 90.1 s^−1^, the particle size range of concrete fragments with the highest proportion is 10~20 mm, accounting for 37%. When the particle size is less than 10 mm, the proportion of each particle size range increases with an increase in the strain rate. It can be seen that the higher the strain rate is, the higher proportion of small particles in the concrete and the more new surfaces formed after the concrete failure.

**Figure 7:**
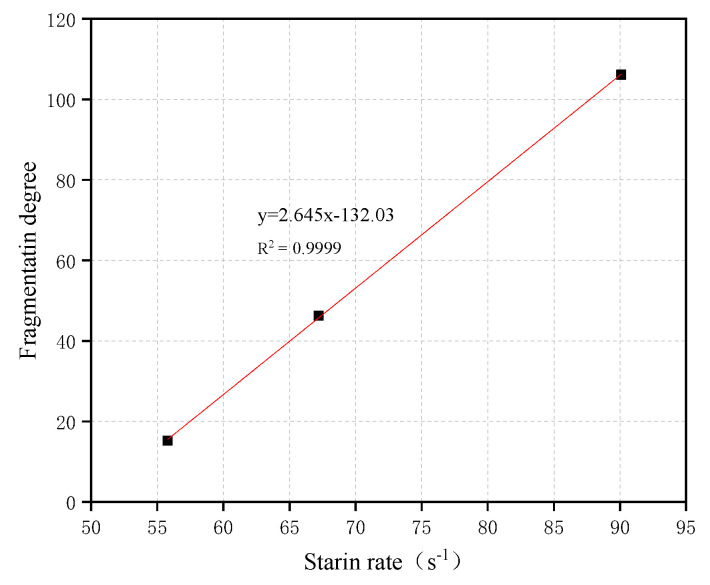
The relationship fragmentation degree and strain rate.

To more intuitively describe the fragmentation distribution of concrete specimens after being impacted, fragmentation degree I is defined to express the degree of fragmentation of concrete specimens damaged by impact loading.

(8) $$I=\sum \frac{r_{i}}{d_{i}}$$

where $d_{i}$ is the average size of the fragments retained in the sieves of different apertures and $r_{i}$ is the percentage of the mass of the fragments corresponding to $d_{i}$. The variation in fragmentation degree with strain rate is shown in Figure 7. It shows that the higher the strain rate is, the higher the fragmentation degree of concrete, which is basically a linear relationship. The higher the loading rate is, the smaller the average size of the sample fragments, the larger the new surface area formed, and the greater the energy used for fracture. The fracture energy of concrete is considered as a kind of material property that does not change with the change in loading rate [22]. Thus, the higher the strain rate is, the larger the new surface area formed by concrete failure, the more energy consumed by fracture, the more energy absorbed from the loading device, the larger the area enclosed by the stress–strain curve of concrete, and the higher the dynamic compressive strength.

### 3.3. Process of Energy Evolution

**Figure 8:**
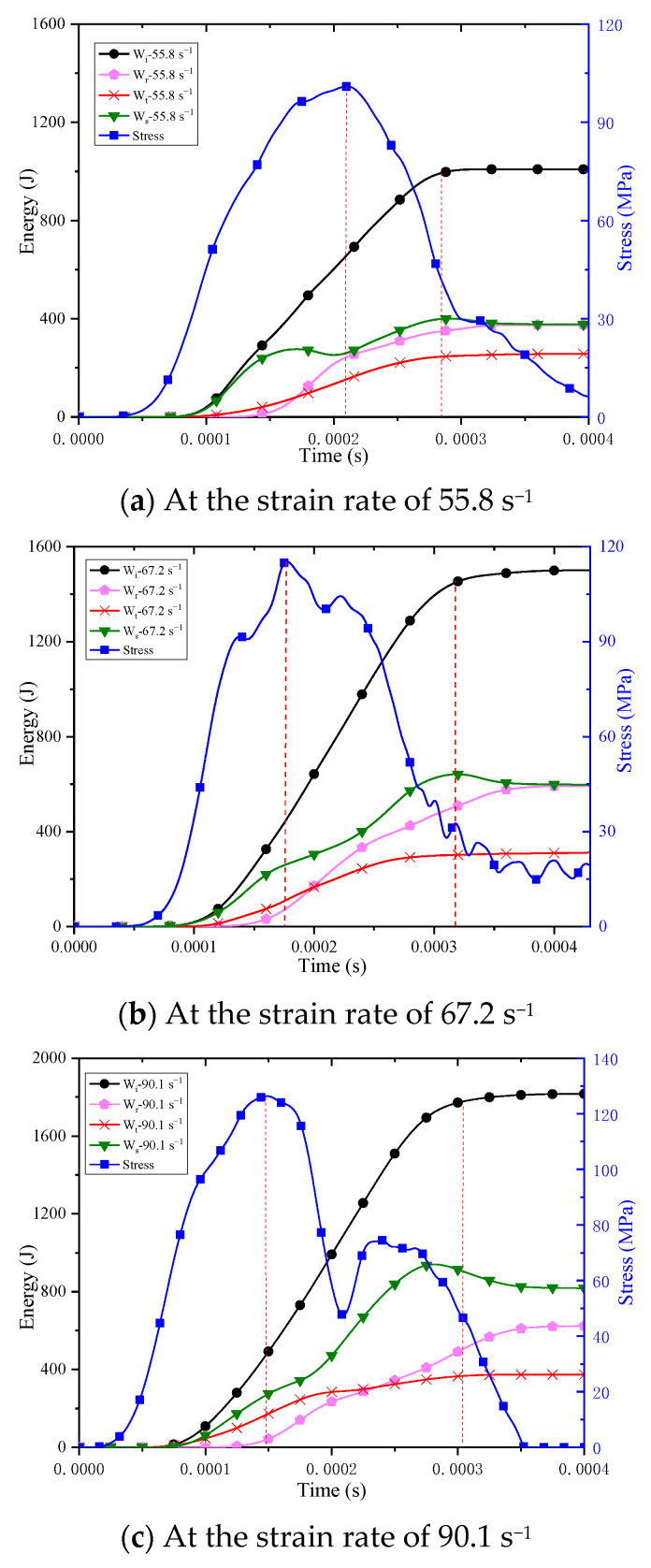
Time curves of energy and stress of concrete under different strain rates.

Under the condition that the distance between the strain gauges on the incident bar and transmission bar and the end face of the specimen and the stress wave velocity in the bar are known, the change process of the incident wave, reflected wave and transmission wave at the end face of the specimen can be calculated. Combined with Formulas (4)~(7), the change process of the incident energy, reflected energy, transmission energy and consumption energy can be obtained. The energy change of concrete when the strain rate of 67.2 s^−1^ is shown in Figure 8b, and the time curve of stress of concrete is also attached to Figure 8 to better analyze the relationship between the energy change process and the stress change process of concrete. According to the curve in Figure 8, it can be considered that the energy conversion process of concrete from loading to failure can be divided into four stages: (1) The stage of energy storage—at the initial stage of loading, most of the incident energy is transmitted into the concrete and absorbed by the concrete, which is converted into the elastic strain energy of the concrete and part of the energy consumed for microcrack propagation. Furthermore, a small part of the incident energy is transmitted from the concrete to the transmission bar and converted into transmission energy, while a small part of the incident energy undergoes interface reflection and is converted into reflected energy. At this time, the conversion rate of incident energy to concrete is high, and the stress of concrete rises sharply. When the incident energy reaches 26.36% of its peak, the concrete reaches the peak stress. The subsequent incident energy cannot affect the dynamic strength of concrete but will increase the failure degree of concrete. Combined with the analysis of fragmentation degree in the previous section, it can be considered that an increase in fragmentation degree of concrete increasing with the increase in strain rate is not only due to the increase in concrete strength which leads to higher internal energy storage, but more importantly caused by the excessive incident energy after the concrete reaches the peak stress. (2) In the stage of elastic strain energy release—after reaching the peak stress, the concrete stress decreases, the rising amplitude of consumption energy decreases, the rising amplitude of reflected energy increases and the rising amplitude of transmission energy also increases to some extent. When the amplitude of change in incident energy is relatively stable, the increase in the rising amplitude of reflected energy and transmission energy is due to the elastic strain energy in concrete presented as release, after the concrete reaches the peak stress. Part of the elastic strain energy stored in the specimen is used for crack propagation and development, and the other part acts on the end face of the specimen and bar, which is converted to reflected energy and transmission energy, leading to an increase in the rising amplitude of reflected energy and transmission energy and a decrease in the ability of concrete to absorb energy. This law is consistent with the view of scholars that the elastic strain energy shows release after peak stress [20]. In addition, the stress of concrete decreases slowly after reaching the peak stress, and its mechanical performance is similar to that of plasticity, but its mechanism is different from that of plastic material. Because the decrease in concrete stress is directly related to the absorption and release of energy, under dynamic loading, due to the high loading rate, the energy absorbed by concrete cannot be released rapidly, transforming into reflected energy and transmission energy, which leads to the slow decline in concrete internal stress. (3) In the stage of crumbing—after experiencing the stage of slow stress decline, the stress of concrete decreases rapidly, while the rising amplitude of consumption energy increases again. The incident energy at this stage is mainly used for crack propagation in concrete, and the degree of crack propagation increases sharply. The elastic strain energy stored in the concrete is mainly applied to crack propagation. Therefore, the rising amplitude of consumption energy is increased, and the rising amplitude of reflected energy and transmission energy is reduced. Because of the rapid destruction of concrete, the ability of energy to propagate from the specimen to the transmission bar also decreases rapidly, and the transmission energy gradually tends to be stable. (4) Stage of residual energy—as the rising amplitude of incident energy decreases and gradually stabilizes. However, due to the high rate of loading, there is still part of the energy that can be released inside the specimen, which leads to the further crushing of the concrete, and at the same time, there is part of the energy transmitted to the incident bar and converted to reflected energy. Therefore, the consumption energy will experience a small reduction stage after reaching the peak.

According to the energy variation process of concrete under impact loading, the mutual conversion process of energy can reflect the mechanical properties of concrete, among which the change in consumption energy variation amplitude can directly reflect the four stages of concrete from being loaded to failure.

Compared with the variation process of incident energy, reflected energy, transmission energy, consumption energy and stress change with time under three strain rates, as shown in Figure 8. The higher the strain rate, the greater the slope of the curve of incident energy, which means that the higher the conversion rate of incident energy is, the earlier the concrete reaches peak stress; however, the incident energy stress decreases with the increase in strain rate at the peak stress moment. It can be seen that an increase in the incident energy rate will reduce the incident energy required for concrete to achieve its strength. From the perspective of engineering, it can be considered that under the same energy input, the higher the loading rate is, the more easily failure occurs in concrete. Under different strain rates, the consumption energy at the peak stress moment of concrete increases with the increasing strain rate but grows slowly. Combined with the incident energy at the peak stress moment, it can be found that the higher the strain rate is, the less incident energy required and the higher the energy absorbed by the concrete. This indicates that the higher the incident energy is, the higher the energy absorption efficiency of concrete is, and the easier it is to reach the energy required at the peak stress moment.

Before the peak stress, under different strain rates, the variation laws of the time-history curve of reflection energy are similar, and the values of reflection energy are close. At the initial stage of loading, the change in reflection energy is less affected by the strain rate, but the increasing amplitude of reflection energy after peak stress increases with the increase in loading rate. This shows that the increasing-amplitude of the reflection energy does not increase immediately with increasing strain rate and has a hysteresis effect. This effect leads to the enhancement of the energy storage capacity in concrete, and the extra energy input into concrete is not released in time, thus enhancing the strength of concrete.

## 4. Conclusions

According to the characteristics of concrete compressive strength increasing with strain rate, dynamic compression tests of concrete under different strain rates have been carried out. From the perspective of energy, the mechanism of concrete dynamic compressive strength improvement is studied, and the following conclusions are given:

The higher the strain rate of concrete loading, the greater the degree of fragmentation, the smaller the size of the fragments, the larger the new surface area formed after the crushing, and the greater the energy used for fracture. The higher the strain rate is, the higher the probability of crack propagation that the cracks pass directly through the aggregate is, and the more regular the fragments are, which are close to cuboid. Since it takes more energy for crack propagation through the aggregate than along the interface, the increase in the dynamic compressive strength of concrete is related to the increase in consumption energy.

By comparing the energy conversion process with the concrete failure process, it is found that the conversion process of each energy in the test can reflect the mechanical properties of concrete in the process of load-bearing failure, in which the change in the variation amplitude of consumption energy corresponds to the four stages of loading failure.

According to the variation process of reflected energy under different impact velocities, the mechanism of dynamic strength increase in concrete is proposed: the variation amplitude of reflected energy that changes with the strain rate before peak stress is small, and it can be considered that the increased amplitude of reflected energy lags behind the increased amplitude of incident energy. As a result, the energy stored in concrete is not released in time through reflected energy, resulting in an increase in energy storage and an increase in dynamic strength.

## Figures and Tables

**Table 1:** The mix proportions of concrete.

Cement (kg/m^3^)	Water (kg/m^3^)	Sand (kg/m^3^)	Aggregates (kg/m^3^)	Water-to-Cement Ratio	Aggregates Size (mm)	Strength Grade
383.88	153.55	782.3	1080.29	0.4	5-20	C30

## Data Availability

The dataset used to support the findings of this study is available from the corresponding author upon request.
